# A Network Analysis of Inner Strength Among University Students with Borderline Personality Disorder Symptoms

**DOI:** 10.3390/ejihpe16020019

**Published:** 2026-01-31

**Authors:** Yuting Song, Justin DeMaranville, Kanyarat Khattiya, Kelvin C. Y. Leung, Nahathai Wongpakaran, Tinakon Wongpakaran

**Affiliations:** 1 Mental Health Program, Multidisciplinary and Interdisciplinary School (MidS), Chiang Mai University, Chiang Mai 50200, Thailand; yuting_s@cmu.ac.th (Y.S.); justinross.dem@cmu.ac.th (J.D.); nahathai.wongpakaran@cmu.ac.th (N.W.); 2 Mental Health and Psychiatry Services, Maesai Hospital, Chiang Rai 57130, Thailand; jammiitennyson@gmail.com; 3 Faculty of Medicine and Health, The University of Sydney, Sydney 2006, Australia; kelvin.leung1@health.nsw.gov.au; 4 Department of Psychiatry, Faculty of Medicine, Chiang Mai University, Chiang Mai 50200, Thailand

**Keywords:** borderline personality disorder, inner strength, network analysis, university student, protective factors, positive psychology, loving-kindness, Buddhist psychology, BPD symptoms, gender differences

## Abstract

Inner strength is increasingly recognized as a protective factor in mental health, but its structure and dynamics remain underexplored, particularly in individuals with borderline personality disorder (BPD) symptoms. This study applied network analysis to investigate the complex relationships among inner strengths in individuals exhibiting BPD symptoms, aiming to identify core and bridging strengths that could inform targeted interventions. The sample comprised 346 Thai university students (25.4% males, 74.6% females; mean age = 21.60 ± 2.24 years) who screened positive for BPD symptoms using the Screening Instrument for Borderline Personality Disorder. Network analysis revealed that inner strengths formed an interconnected system with both core and peripheral features. The strongest association was observed between generosity and loving-kindness. A cognitive behavioral cluster comprising perseverance, wisdom, and determination also emerged. Centrality analysis identified loving-kindness as the most influential node in terms of direct connections, while equanimity exhibited the highest bridge centrality. Generosity and determination demonstrated the greatest expected influence. A negative link between truthfulness and equanimity highlighted a potential conflict between absolute honesty and inner balance in this population. Notably, exploratory analyses by gender revealed distinct network structures. In males, determination, wisdom, and precept emerged as central strengths; however, results for males should be interpreted with caution due to sample size. In females, perseverance, loving-kindness, and meditation were the most influential. These gender-specific patterns suggest that targeting different core strengths according to gender may enhance the effectiveness of interventions for individuals with BPD symptoms. Overall, the findings suggest that fostering specific inner strengths, particularly equanimity, loving-kindness, and those identified as central for each gender, may enhance psychological resilience and inform more tailored intervention strategies for BPD.

## 1. Introduction

Borderline Personality Disorder (BPD) is a complex psychiatric condition characterized by affective instability, impulsive behavior, identity disturbance, and unstable interpersonal relationships (Leichsenring et al., 2011, 2023; Morey & Zanarini, 2000). Its prevalence is notably higher among Thai university students (6.4%) than in the general population (Biskin, 2015; Eaton & Greene, 2018; Lohanan et al., 2020).

BPD symptoms often emerge and persist during emerging adulthood (Videler et al., 2019), a distinct developmental stage bridging adolescence and full adulthood (approximately ages 18–29), characterized by identity exploration, instability, self-focus, and a sense of being “in-between”, which includes the college years (Arnett, 2000). University students occupy a particularly vulnerable position within this life stage. During this stage, They are confronted with critical developmental tasks, including the establishment of autonomy, identity consolidation, and the formation of stable intimate relationships (La Rosa et al., 2025). However, the inherent features of BPD intertwine with these developmental challenges, potentially exacerbating adaptive difficulties for individuals (Cano et al., 2022; Jia et al., 2022; Meaney et al., 2016; Mueller, 2023; Videler et al., 2019; Wongpakaran et al., 2021). This overlap highlights the importance of identifying protective factors to foster resilience in this high-risk group.

Traditionally, research has examined protective factors like resilience and self-compassion (Kouklidou et al., 2025; Southward et al., 2023). Recently, the broader construct of inner strength—a multidimensional psychological resource that promotes stability and growth under stress has gained attention (Lundman et al., 2010; Masten, 2001). From a positive psychological perspective, inner strength constitutes cultivable traits that serve as a foundation for well-being (Csikszentmihalyi & Seligman, 2000). In Thai society, this concept aligns closely with the Theravāda Buddhist framework of the “ten perfections” (Pāramī), including qualities: Truthfulness, Perseverance, Wisdom, Generosity, Five Precepts, Meditation, Tolerance, Equanimity, Determination, and Loving-kindness (Buddhaghosa, 2020; Wongpakaran et al., 2020). These culturally resonant virtues have shown promise in mitigating BPD symptoms by enhancing emotion regulation and reducing rumination, reducing self-injurious behaviors and moderate the effects of neuroticism and perceived stress (Deng et al., 2025; Fernandes et al., 2022; Glass et al., 2024; Keng & Tan, 2018; Lam & Seiden, 2020; Sripunya et al., 2024; Tsai & Morissette, 2022; Wongpakaran et al., 2021).

However, inner strength is not a single trait but a system of interrelated components. Understanding how these components interact and support each other is essential for developing effective interventions. Network analysis provides a powerful methodological framework for this purpose. Unlike traditional latent variable models, network analysis conceptualizes psychological constructs as systems of directly interacting nodes (Borsboom & Cramer, 2013). This approach allows researchers to visualize the structure of a construct, identify the most central or influential node, and detect bridging node that connect different clusters, which is crucial for identifying precise intervention targets (Borsboom et al., 2021; Hevey, 2018; McNally, 2021). While network analysis has been applied to BPD symptom networks (Richetin et al., 2017), its application to the network structure of protective inner strengths in individuals with BPD symptoms remains unexplored.

**Figure 1:**
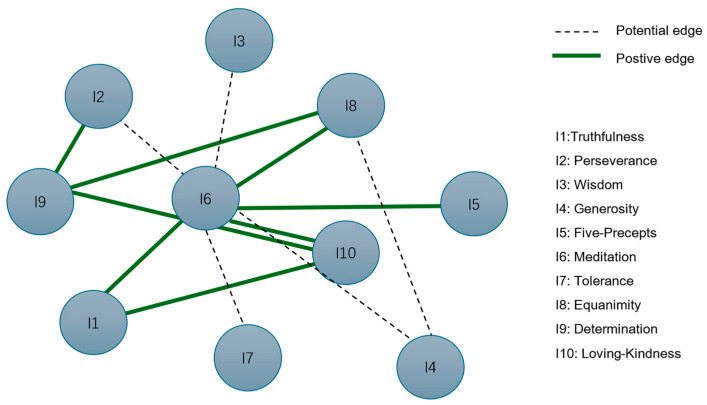
Conceptual model illustrates the hypothesized relationships between inner strengths.

This study seeks to address a significant gap in the literature by applying network analysis to explore the interconnectedness of ten Buddhist inner strengths in Thai university students who screen positive for BPD symptoms. Drawing from both existing research and Vipassanā traditions, Buddhist teachings emphasize that the fulfillment of all ten strengths is essential for spiritual development and the attainment of enlightenment. Central to this process are meditation (bhāvanā) and loving-kindness (mettā), which are believed to serve as foundational or hub strengths that facilitate the development of the other eight (Thavaro, 1982). Given the lack of prior studies investigating the network structure of inner strengths specifically in individuals with BPD symptoms, this study adopts the Buddhist theoretical framework to guide its hypothesis. Specifically, Figure 1 shows we propose that meditation and loving-kindness will emerge as the most central and influential strengths within the network. These strengths are expected to serve as key nodes, exerting a strong direct influence on other inner strengths, in line with traditional Buddhist practice and prior literature.

## 2. Materials and Methods

### 2.1. Participants and Data Collection

This study used a cross-sectional design and network analysis as a secondary analysis of an existing dataset. The original research, “Association Between Pets and Mental Health in University Students with Borderline Personality Disorder Symptoms”(Khattiya et al., 2026), was conducted at Chiang Mai University, Thailand, with approval from the university’s Ethics Committee (Approval No. PSY-2566-0502). Data were collected from November 2021 to August 2022, with all participants providing written informed consent. Recruitment was via online and physical advertisements at university campuses and psychiatric clinics. Of 1244 students contacted, 693 were excluded (7 declined consent; 686 scored ≤ 7 on the SI-Bord). Among 551 assessed for eligibility, 205 were further excluded (26 duplicate responses; 179 declined participation), resulting in 346 students with SI-Bord scores ≥ 7 included in the original analysis. For the present study, we addressed a new research question by extracting the inner strength variable from this database.

### 2.2. Instruments

#### 2.2.1. Screening Instrument for Borderline Personality Disorder (SI-Bord)

SI-Bord is a 5-item self-report tool based on the core features of BPD from the Diagnostic and Statistical Manual of Mental Disorders, 5th Edition (DSM-5). It assesses five domains: fear of abandonment, unstable interpersonal relationships, identity disturbance, self-harm behavior, and affective instability. Items are rated on a 4-point Likert scale ranging from 0 (never) to 3 (very often), with total scores ranging from 0 to 15. In this study, a cutoff score of ≥7 was adopted to optimize screening sensitivity, yielding 75.0% sensitivity and 73.1% specificity in a Thai university student sample. The internal consistency reliability (Cronbach’s α) of the scale was 0.76 (Lohanan et al., 2020).

#### 2.2.2. Inner-Strength-Based Inventory (I-SBI)

I-SBI was developed based on the Buddhist psychological concept of the “Ten Perfections”. The tool aims to measure ten positive inner qualities: Truthfulness, Perseverance, Wisdom, Generosity, Five Precepts, Meditation, Tolerance, Equanimity, Determination, and Loving-kindness. Each of the ten items represents one dimension. Responses are recorded on a 5-point scale with higher scores representing higher levels of inner strength. Each dimension is assessed by a single item. Each dimension is assessed by a single item, such as “I regularly keep promises even for trivial matters” (Truthfulness), “I think I am diligent, hardworking, and do extra work” (Perseverance), “I always think through my decisions every time before I carry them out” (Wisdom), “I always offer help; if it is beyond my capabilities, I will try to find alternatives for them” (Generosity), “I always follow the Moral virtues. As I can remember, I have never broken them before” (Five Precepts), “I meditate every day, at a certain time including some other time available” (Meditation), “I try to make my facial and verbal expressions appear normal so that everything can carry on as it should be” (Tolerance), “I can get over losses or separations in a shorter time compared with others” (Equanimity), “I have my life goals and I set targets for everything that I do” (Determination), and “I always feel sympathetic for everyone even if I do not like him or her” (Loving-kindness). Psychometric evaluation supported the scale’s unidimensionality, good item fit, and reliability, with a person separation reliability of 2.45, person reliability coefficient of 0.86, and item reliability of 0.99 (Wongpakaran et al., 2020). However, internal consistency metrics such as Cronbach’s α for the i-SBI cannot be calculated, as each dimension is measured using a single item.

### 2.3. Statistical Analyses

#### 2.3.1. Descriptive Statistical Analysis

Data preprocessing and descriptive statistical analyses were conducted using SPSS (v.27.0) (Meyers et al., 2013). A small amount of missing data was handled using the Expectation–Maximization algorithm (Pigott, 2001). Descriptive statistics were calculated for demographic variables (age, gender, and years of study) and the ten dimensions of inner strength.

#### 2.3.2. Network Analysis Overview

Network analysis is an innovative statistical approach that conceptualizes psychological constructs as systems of interacting variables (nodes) rather than as isolated factors influenced by latent variables. In this framework, each node represents a distinct dimension of inner strength, and edges between nodes represent unique associations (partial correlations) after controlling for all other variables in the network. This method enables the identification of direct relationships among strengths, highlights the most central and influential characteristics, and reveals the overall connectivity pattern within the system.

#### 2.3.3. Network Estimation and Visualization

A regularized partial correlation network was estimated using the Extended Bayesian Information Criterion graphical LASSO (EBICglasso) method, as implemented in the qgraph package (Epskamp et al., 2018). EBICglasso was selected because it is especially well-suited to psychological research involving continuous or ordinal data, such as the inner strength dimensions assessed in this study. Unlike unregularized correlation or partial correlation networks, EBICglasso applies a regularization technique that shrinks weak and potentially spurious connections towards zero, resulting in a sparse and interpretable network structure. This approach reduces the risk of overfitting and highlights only the most robust, meaningful associations between variables, thereby facilitating clearer insights into the core and bridging inner strengths among university students with borderline personality disorder symptoms.

Prior to network estimation, all variables were standardized implicitly through the correlation-based estimation procedure, ensuring comparability across dimensions. The network layout was generated using the spring algorithm. To aid interpretability in visualizations only, we applied a minimum edge weight parameter (minimum = 0.5) and edge weight threshold set at |r| > 0.1 were applied. These threshold is commonly applied in psychological network analysis to balance the retention of meaningful associations against the exclusion of weak associations that may arise from random error or multiple comparisons, (Epskamp et al., 2018). Importantly, these parameters affected visual clarity alone and had no influence on the estimation, edge selection, or any subsequent statistical analyses, all of which were based on the full weighted network.

In the final undirected network, each node represented a dimension of inner strength, while edges indicated the strength and direction of the partial correlation between two nodes. Green edges represented positive correlations, red edges negative correlations, and edge thickness corresponded to correlation strength (Fried & Cramer, 2017).

#### 2.3.4. Community Structure Detection

Community detection (also known as clustering) was performed using the Louvain algorithm. Community structure within the network was identified using the Louvain algorithm from the igraph package (V.2.2.1) (Epskamp et al., 2018). The Louvain method clusters nodes into communities based on the maximization of modularity, such that nodes within the same community are more densely interconnected with each other than with nodes outside the community.

In the present network, only a very limited number of negative edges were observed, and only one negative edge reached statistical significance. Including such sparse negative connections in modularity-based community detection algorithms may lead to unstable or difficult-to-interpret community solutions (Esmailian & Jalili, 2015). Therefore, solutions based on positive edge weights were used for interpretation and visualization in the final network model. This approach is commonly adopted in network analyses when negative edges are rare, as it allows communities to reflect clusters of mutually reinforcing components (Breiger et al., 1975).

#### 2.3.5. Centrality Analysis

In addition to reporting node strength, we included expected influence as a centrality index in our network analysis. Node strength, defined as the sum of the absolute edge weights connected to a node, is widely regarded as a robust measure of node importance within psychological networks. Expected influence extends this by accounting for the direction of connections, thus capturing both positive and negative relationships (Robinaugh et al., 2016). This is particularly relevant in our analysis, given the presence of both supportive and antagonistic associations among inner strengths.

We computed four centrality indices, Strength, Closeness, Betweenness, and Expected Influence, calculated via qgraph (V.1.9.8) and networktools (V.1.6.0), to evaluate node importance from multiple perspectives (Castro et al., 2019; Epskamp et al., 2018). Centrality values were standardized (z-scores) and illustrated in a centrality profile plot, helping to identify which inner strengths play the most influential roles in the network.

#### 2.3.6. Network Stability and Accuracy

Finally, network stability and accuracy were evaluated using the bootnet (V.1.6) (Epskamp et al., 2018). Nonparametric bootstrapping with 1000 samples generated 95% confidence intervals for edge weights; edge weights were considered stable and interpretable if the confidence intervals were relatively narrow and did not include zero. The robustness of centrality estimates, including strength, closeness, betweenness, and expected influence, was evaluated using case-dropping bootstrapping. The correlation stability (CS) coefficient was calculated for each index to quantify stability; according to established guidelines, a CS coefficient above 0.25 is considered acceptable, while a value above 0.50 indicates good stability. Centrality indices below the 0.25 threshold should be interpreted with caution.

## 3. Results

### 3.1. Descriptive Statistics

The study sample consisted of 346 university students recruited for the analysis. Key demographic characteristics including age, gender, and academic year, are summarized in Table 1.

Table 2 shows descriptive statistics for the ten inner strength qualities. The mean scores ranged from 1.49 to 3.48. Specifically, Generosity recorded the highest mean score, whereas Meditation yielded the lowest. The remaining variables had mean scores ranging from 2.41 to 3.23. The total score for the ten inner strengths had a mean of 28.72 (SD = 5.73), with observed scores ranging from 10 to 41.

An examination of skewness and kurtosis indicated that the score distributions for most variables approximated normality, though Meditation demonstrated a pronounced positive skewness (skewness = 1.98) and a high kurtosis value (kurtosis = 4.58). Given that the network constructed in the present study is based on partial correlations, the estimation is relatively robust to deviations from normality in the variable distributions. Subsequent network stability analyses, such as bootstrap confidence intervals, further indicated that the estimates of the primary edge weights were reliable. Accordingly, the non-normal distribution of the meditation item is scarcely possible to have exerted a decisive influence on the core structure of the network.

Table 3 shows The correlation matrix indicates that inner strengths tend to cluster together, suggesting these qualities reinforce each other within individuals. Females report higher levels of Generosity, Loving-kindness, and adherence to the Five Precepts, possibly reflecting sociocultural influences. Meditation practice appears linked to several strengths but is largely independent of age or gender. Overall, the pattern underscores the interdependent nature of inner strengths, rather than their development being driven by demographic factors.

Correlation analysis also revealed that female gender was significantly associated with higher levels of generosity, loving-kindness, and adherence to the Five Precepts. Relationships among the different dimensions of inner strength were generally positive and significant, suggesting a cohesive cluster of strengths within individuals. However, there were no significant correlations between age or year of study and any of the inner strength dimensions, indicating that these qualities remain relatively stable across the university years in students with BPD symptoms. Further analysis for the effect of gender in network structure was conducted.

### 3.2. Network Analysis

In the regularized partial correlation network constructed from the ten nodes derived from levels of inner strength, the network comprised 45 potential edges, of which 17 were significant (based on 1000 bootstrap samples, 95% confidence intervals not including zero). The mean weight of these edges was 0.157. Among all significant edges, 16 exhibited positive weights, with a mean of 0.174, whereas one edge exhibited a negative weight (mean = −0.115).

**Figure 2:**
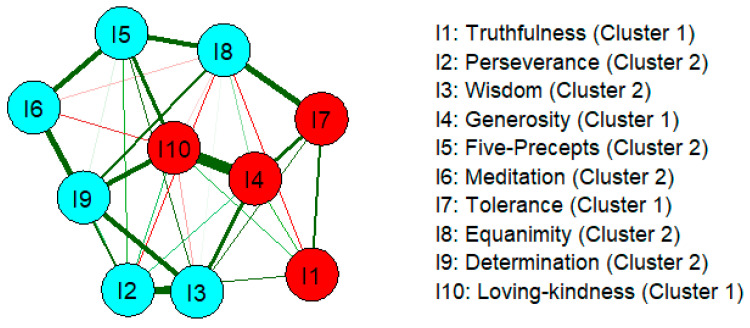
Network of Inner Strength in BPD. Each circle (node) represents the inner strength dimension, The red circles represent cluster 1, and the blue circles represent cluster 2. While lines (edges) indicate relationships between nodes. These relationships are represented by weight values in the network that are based on partial correlations. Thicker and darker edges signify stronger relationships. Green edges denote positive relationships, and red edges represent negative relationships.

Figure 2 presents the network structure visualized from the original partial correlation matrix. The strongest connection was observed between Generosity and Loving-kindness (r = 0.399), followed by Perseverance and Wisdom (r = 0.318), and Wisdom and Determination (r = 0.295). A unique weak negative edge was identified between Truthfulness and Equanimity (r = −0.069).

The complete edge weight partial matrix can be found in Table S3 of the Supplementary Materials.

Community detection via the Louvain algorithm revealed two prominent communities (clusters), distinguished by node color: Cluster 1 (red) comprises Truthfulness, Generosity, Tolerance, and Loving-kindness, while Cluster 2 (cyan) comprises Perseverance, Wisdom, Five Precepts, Meditation, Equanimity, and Determination. Within each community, nodes exhibit more densely interconnected patterns, indicating stronger within-community relationships.

**Figure 3:**
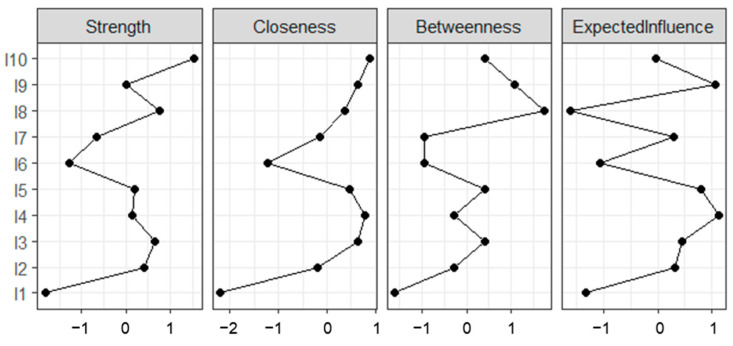
Centrality indices. All centrality values are z standardized. I1 = Truthfulness; I2 = Perseverance; I3 = Wisdom; I4 = Generosity; I5 = Five Precepts; I6 = Meditation; I7 = Tolerance; I8 = Equanimity; I9 = Determination; I10 = Loving-Kindness.

The results of the centrality analysis are presented in Figure 3. Unlike the assumption, Loving-kindness (z = 1.069) and Equanimity (z = 1.58) demonstrated the highest centrality strength, indicating these strengths hold the strongest direct connections with other inner strengths within the network. Bootstrap stability analyses indicated that strength and closeness centrality exhibited limited stability; therefore, these indices should be interpreted with caution. Both strength and expected influence centrality metrics were computed for all nodes. While the overall patterns were generally consistent between the two metrics, expected influence allowed for a more nuanced understanding of how certain inner strengths, such as truthfulness and equanimity, may exert opposing influences within the network. Generosity (expected influence z = 0.897) and Determination (expected influence z = 0.882) exhibited higher expected influence centrality, suggesting that these dimensions may have a greater overall impact across the network when considering both positive and negative associations.

In addition, Equanimity (Betweenness Centrality = 1.727) had the highest bridge centrality compared with other nodes, emphasizing its key role as a connector or bridge between different parts of the network. Closeness centrality values were generally low for all nodes, suggesting that there is no single strength that notably shortens the path between other strengths in the network. Overall, these findings suggest a relatively distributed network structure with no distinctly central ‘hub,’ and warrant careful interpretation given the instability of some centrality measures (See Supplementary File S1).

### 3.3. Exploratory Network and Centrality Analyses by Sex

To examine gender differences, we estimated the network structure and centrality of inner strengths separately for male and female students with BPD symptoms. For the male subgroup, EBICglasso produced an empty network, likely reflecting insufficient sample size and strong regularization. Using unregularized partial correlations yielded a fully connected network with nonzero centrality for all variables, but these edges may be unreliable because unregularized estimates are prone to overfitting in small samples. Therefore, findings for the male subgroup should be interpreted with caution (see Supplementary File S2 for details output).

### 3.4. Network Structure and Key Associations

For female students, the strongest positive associations were found between Perseverance and Wisdom, and between Loving-kindness and Generosity. Other robust connections included Precept and Meditation as well as Perseverance and Determination. Notably, there was a negative link between Truthfulness and Equanimity, highlighting a potential internal tension in the expression of these qualities among females.

Among male students, the strongest connection was observed between Wisdom and Generosity, suggesting these strengths are closely linked in this group. Additional prominent positive associations included Perseverance with Truthfulness, and Precept with Meditation. Negative associations were identified, such as between Tolerance and Precept, and Perseverance and Equanimity, indicating areas of potential internal conflict or divergence among certain strengths in males.

### 3.5. Centrality and Influence of Strengths

Centrality analyses revealed distinct patterns of influential nodes by sex: In females, Perseverance was the most central and influential strength, followed by Loving-kindness and Meditation. Tolerance also showed notable bridging capability. Consistent with the male network, Truthfulness was the most peripheral in females, while Wisdom was less central compared to its role among males.

In males, Precept and Wisdom emerged as the central hubs in the network, serving as key bridges among other strengths. Determination demonstrated the highest expected influence, suggesting changes in this strength could have widespread effects within the male network. In contrast, Truthfulness and Equanimity were peripheral, carrying less influence.

In sum, comparing partial correlation networks of Buddhist inner strengths by gender reveals distinct patterns. For females, strengths like perseverance, tolerance, and loving-kindness hold the highest centrality, indicating they are crucial hubs. In contrast, among males, wisdom and five precepts are most central, suggesting different dynamics in the inter-relationships of Buddhist qualities. Furthermore, several pairwise relationships, such as between generosity and other strengths, vary in strength and sign by gender, highlighting gender-specific mechanisms in the web of inner strengths

**Figure 4:**
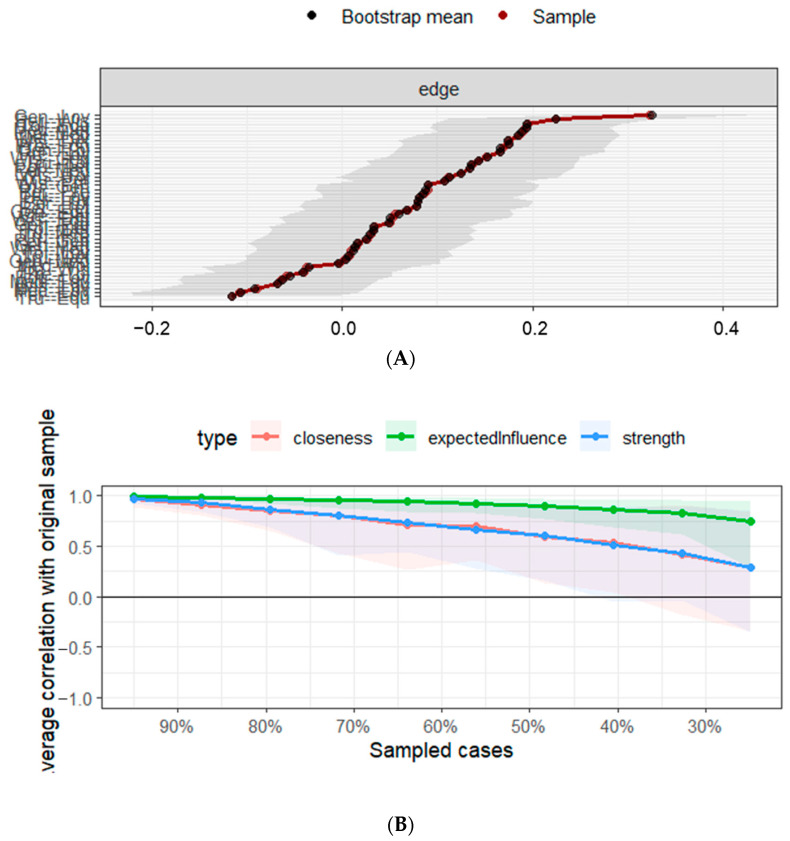
(**A**). Accuracy of the edge-weight estimates. Black dots represent the bootstrap mean of edge weights, and red dots indicate the original sample estimates. Gray areas denote the 95% bootstrap confidence intervals for each edge. Edge weights are ordered from smallest to largest. (**B**). Stability of the central indices. The *x*-axis represents the proportion of cases retained in the subsample, and the *y*-axis indicates the correlation between centrality estimates obtained from the original sample and those from subsamples. Lines represent different centrality measures (strength, expected influence, and closeness), with shaded areas indicating variability across bootstrap samples.

Figure 4A displays the bootstrapped 95% confidence intervals (CIs) for the estimated edge weights in the network. Most edge weights have relatively narrow confidence intervals, suggesting that the strength of the associations between nodes is estimated with good precision and stability. The CIs for most edges do not include zero, indicating that these connections are statistically robust and unlikely to be spurious. This provides further confidence in the interpretability of the network’s detected edges.

Figure 4B shows that expected influence (green line) exhibited the highest stability, with a correlation stability (CS) coefficient well above the recommended threshold of 0.50 (CS = 0.595), indicating that this centrality measure remains robust even when a substantial proportion of cases is removed. In contrast, strength (blue line) and closeness (red line) demonstrated lower stability, with CS coefficients approaching or only slightly exceeding 0.25. This suggests that these indices are less robust and therefore should be interpreted with caution.

The stability of the network estimates was evaluated primarily on the full sample to provide an overall assessment of the robustness of the network structure and centrality indices. Subgroup analyses by sex were also conducted, but these should be interpreted with caution, especially for the male subgroup, which had a smaller sample and therefore more limited stability for centrality measures.

Expected influence was the only centrality metric that showed reliable stability. In the female subgroup, Perseverance had the highest expected influence, followed by Loving-kindness and Meditation. In the male subgroup, Determination showed the highest expected influence; however, this result should be treated cautiously because the subgroup-specific network estimates were less stable.

## 4. Discussion

This study is the first to employ a network analytic approach to explore the dynamic associative structure of inner strengths among individuals with BPD symptoms. Although previous studies have demonstrated the protective role of inner strength in mental health, most have focused on examining single strengths or they have treated inner strength as a unidimensional construct (Glass et al., 2024; Sripunya et al., 2024). By revealing the complex network of interactions among these strengths, the present study offers a novel perspective for understanding the positive psychological structure underlying BPD.

The network analysis revealed that inner strengths do not exist in isolation, but they form an integrated system characterized by both core and peripheral structures. Among all nodes, the strongest association was observed between Generosity and Loving-kindness, consistent with Buddhist teachings that regard dāna (generosity) and mettā (loving-kindness) as complementary paths of practice, supported by prior theoretical assumptions about their synergistic function (Ariyabuddhiphongs, 2016; Dorjey, 2018). In addition, strong connections between Perseverance, Wisdom, and Determination formed a cognitive behavioral cluster that reflects the inherent unity of cognitive resources and behavioral motivation in goal-directed functioning. These communities may reflect underlying domains of psychological resources, such as interpersonal virtues and self-regulatory or contemplative strengths, which may function together within the psychological network of individuals with BPD. These patterns highlight a shared psychological structure and potential cooperation among strengths, though the direction or causal nature of these relationships cannot be inferred from the current cross-sectional analysis.

From the centrality analysis, Loving-kindness exhibited the highest strength centrality, indicating that it had the most direct connections within the network and served as a central hub of the system. While this finding underscores the structural prominence of loving-kindness within the network, its suitability as a clinical intervention target cannot be concluded from these cross-sectional results alone. As one of the “Four Immeasurable” in Buddhist psychology, loving-kindness has been shown to enhance emotional regulation and interpersonal functioning—core areas of impairment in individuals with BPD (Keng & Tan, 2018). Equanimity demonstrated a distinctive bridge by connecting different clusters of strengths within the network. Although equanimity’s position may be important for understanding the organization of these strengths (Jijina & Biswas, 2022), further research using longitudinal or experimental designs is required to determine its potential role in targeted interventions for emotion regulation.

Notably, the network analytic approach highlights the structural importance of central or “hub” strengths. In network models, central nodes are structurally well-connected, suggesting they are integral parts of the overall associative structure. However, we cannot infer that strengthening these hub strengths, such as loving-kindness and equanimity, would necessarily lead to changes in other areas of the network or broader psychological outcomes, given the limitations of cross-sectional data. While the identification of central strengths can inform future research directions, intervention effects or causal relationships remain to be clarified by longitudinal or experimental studies.

One of the most intriguing findings of our network analysis was the negative association between Truthfulness, operationalized as keeping promises, and Equanimity. While both are generally regarded as positive qualities, their inverse relationship in individuals exhibiting BPD symptoms requires careful interpretation and engagement with the clinical and developmental literature.

From the perspective of borderline pathology, individuals with BPD are known to struggle with emotion regulation and often display a degree of psychological rigidity, particularly around value-driven behavior (Bateman & Fonagy, 2006; Linehan, 1993). Graves et al. (2021) provide important insights by highlighting that deficits in trust and reliability in BPD are not solely interpersonal but are also closely linked to heightened emotional turbulence and internal tension (Graves et al., 2021). They argue that for some individuals with BPD, the enactment of truthfulness—such as consistently keeping promises—can become overly rigid or driven by anxiety. Rather than fostering relational trust or inner harmony, this inflexibility can actually fuel distress when circumstances prevent perfect adherence to these high standards, thereby undermining equanimity.

This interpretation is further supported by developmental literature, which suggests that early disruptions in attachment and trust may lead those with BPD to seek security through uncompromising authenticity or the avoidance of perceived betrayal (Luyten & Fonagy, 2015). Consequently, truthfulness, while adaptive in moderation, may take on maladaptive characteristics when enacted without psychological flexibility or self-forgiveness. This dynamic, in turn, may disrupt the individual’s ability to maintain emotional balance, that is, equanimity, especially in the face of interpersonal stress or internal conflict (Lynch et al., 2006). Additionally, one must consider cultural influences and measurement issues. In the Thai Buddhist context, truthfulness is strongly upheld as a moral value, potentially increasing social desirability bias or internalized expectations for promise-keeping. Thus, the observed negative association might reflect not only maladaptive processes within BPD but also unique cultural tensions between external duty and inner peace.

Among female students with BPD symptoms, perseverance, tolerance, and loving-kindness emerged as the most central Buddhist inner strengths, reflecting adaptive strategies for managing intense emotions and relational stress. These qualities likely support emotional resilience and compassion, both self-directed and toward others, which are crucial for psychological stability in this group. Although females reported higher levels of Generosity and Five Precepts than males, these strengths were less central in the network, suggesting they are less pivotal for coping with BPD-related challenges. This distinction highlights the importance of targeting perseverance, tolerance, and loving-kindness in interventions to build meaningful resilience in female students with BPD symptoms.

The study demonstrated robust network stability and accuracy, with strong CS coefficients for edge weights and expected influence, supporting reliable interpretation. In contrast, strength and closeness centrality were less stable and should be interpreted cautiously. These results support Wongpakaran et al.’s inner strength framework and align with self-regulation theory, showing inner strengths operate as an interconnected system to sustain psychological adaptation, though causality cannot be inferred.

### 4.1. Implications of the Study

The findings offer valuable clinical insights for interventions targeting BPD. Identifying central strengths—such as loving-kindness and equanimity provides an empirical basis for precision-based intervention strategies. Future research should examine whether approaches focused on cultivating loving-kindness and mindfulness, including loving-kindness meditation and mindfulness-based training, offer unique benefits for alleviating BPD symptoms, in alignment with distress tolerance skills emphasized in dialectical behavior therapy.

Generosity and determination, which demonstrate high network influence, may serve as promising targets for behavioral activation interventions, such as structured altruistic activities or goal setting, that could lead to positive changes throughout the inner strength system.

The study’s network analysis also revealed gender differences: key strengths for males included Precept, Wisdom, and Determination, while Perseverance, Loving-kindness, and Meditation were most influential for females. These findings highlight the potential of gender-sensitive, strength-based interventions, such as ethical reflection and mindfulness for males, or perseverance and loving-kindness practices for females, to boost resilience, engagement, and motivation.

However, it should be noted that the analysis of network metrics among male participants may be less stable and reliable, likely due to sample size and gender imbalance. Therefore, interpretations regarding core strengths and intervention priorities for males should be approached with caution and validated in future research with larger, more balanced samples.

Overall, precision interventions tailored to individual and gender-specific strength profiles, while mindful of analytic stability, may complement existing BPD treatments and enhance psychological resilience in university students with BPD symptoms.

### 4.2. Limitations and Future Study

This study has several limitations. Its cross-sectional design limits causal interpretations: longitudinal or experimental studies are needed to clarify network relationships. Some centrality measures (strength and closeness) were less stable, so those findings should be considered preliminary. The exclusive use of self-report data from a screening-based sample of Thai university students with elevated BPD symptoms (not formal diagnoses) may introduce biases and restrict generalizability, particularly to other cultural contexts or clinically diagnosed BPD samples. Incorporating multi-method assessments, clinical samples, and non-BPD groups in future research would improve validity and applicability.

The study also had a gender imbalance, which may influence generalizability of network findings beyond this population. Although only two strengths showed significant gender differences, larger and more gender-balanced samples are needed to validate and generalize results.

The focus on inner strength structure without external variables or control groups limits conclusions. Future work should examine these aspects to further test and expand on the current findings. Finally, a methodological consideration is our use of expected influence as a centrality metric. While we report both strength and expected influence, strength centrality is generally more robust and stable in psychological networks. Expected influence offers value in networks with both positive and negative edges but may be more sensitive to sample characteristics. Our stability analyses supported the robustness of expected influence in this study, but we recommend interpreting its unique contributions with caution and prioritizing strength centrality in line with standard practice. Despite these limitations, this study used network analysis with a large BPD symptom sample, providing new insights into the dynamic interplay of Buddhist inner strengths and supporting positive psychology theories.

## 5. Conclusions

This study is the first to use network analysis to map the interconnected inner strengths of individuals with BPD symptoms, moving beyond traditional unidimensional approaches. The results revealed that inner strengths form an integrated system with core and peripheral structures, in which loving-kindness, perseverance, and tolerance play central roles, especially among females. These strengths appear to act synergistically to support emotional resilience and adaptive coping with BPD-related challenges.

Importantly, the network approach highlighted both positive and negative interactions among strengths, such as the inverse relationship between truthfulness and equanimity, suggesting that certain strengths may sometimes compete or create tension within the psychological system. While strengths like loving-kindness and equanimity showed structural prominence, the cross-sectional nature of the study limits conclusions about causality or direct intervention effects.

Overall, the findings support an inner strength framework emphasizing dynamic, mutually reinforcing psychological resources, and point to the potential benefits of precision, strength-based, and gender-sensitive interventions for individuals with BPD symptoms. Future longitudinal and experimental studies are needed to clarify causal relationships, further validate the network structure, and optimize strength-based therapeutic strategies.

## Figures and Tables

**Table 1:** Sample Characteristics (n = 346).

Characteristic	Category	Value
Age, M ± SD	-	21.60 ± 2.24
Gender, n (%)	Male	88 (25.4%)
	Female	258 (74.6%)
Year of Study, n (%)	1st year	33 (9.5%)
	2nd year	85 (24.6%)
	3rd year	95 (27.5%)
	4th year	75 (21.7%)
	Above 4th year	58 (16.8%)

Note. n = number of participants; M = mean; SD = standard deviation.

**Table 2:** Descriptive Statistics for Inner Strength (n = 346).

Variables	Mean (SD)	Min	Max	Skewness	Kurtosis
Truthfulness	3.20 ± 1.27	1	5	0.18	−1.37
Perseverance	2.41 ± 1.03	1	5	0.61	−0.05
Wisdom	2.95 ± 1.19	1	5	−0.11	−0.80
Generosity	3.48 ± 1.27	1	5	−0.49	−1.08
Five Precepts	2.82 ± 1.22	1	5	0.03	−0.91
Meditation	1.49 ± 0.78	1	5	1.98	4.58
Tolerance	3.17 ± 1.13	1	5	−0.07	−1.05
Equanimity	2.95 ± 1.01	1	5	0.03	−0.64
Determination	3.02 ± 1.11	1	5	0.18	−0.96
Loving-Kindness	3.23 ± 1.23	1	5	−0.45	−1.03
Sum	28.72 ± 5.73	10	41	−0.60	0.08

Note. n = number of participants; M = Mean; SD = Standard Deviation.

**Table 3:** Correlation Matrix of Demographic Characteristics and Inner Strength Dimensions.

Variables	1	2	3	4	5	6	7	8	9	10	11	12	13
1. Gender	-												
2. Age	0.00	-											
3. Year of Study	0.10	0.65 ***	-										
4. Truthfulness	0.08	0.07	−0.07	-									
5. Perseverance	−0.01	−0.00	−0.03	0.19 ***	-								
6. Wisdom	0.11	0.01	0.03	0.07 **	0.32 ***	-							
7. Generosity	0.20 ***	−0.06	0.06	0.19 ***	0.19 ***	0.27 ***	-						
8. Five Precepts	0.13 *	−0.01	−0.05	0.09	0.22 ***	0.23 ***	0.21 ***	-					
9. Meditation	−0.03	−0.07	−0.05	0.08	0.21 ***	0.15 **	0.07	0.25 ***	-				
10. Tolerance	0.08	0.06	0.10	0.18 ***	0.18 ***	0.24 ***	0.28 ***	0.10	0.06	-			
11. Equanimity	−0.10	0.02	0.09	−0.07	−0.02	0.15 **	0.13 **	0.20 ***	0.00	0.22 ***	-		
12. Determination	−0.00	−0.03	0.08	0.09	0.23 ***	0.30 ***	0.21 ***	0.24 ***	0.25 ***	0.17 **	0.18 ***	-	
13. Loving-kindness	0.27 ***	−0.03	0.02	0.18 ***	0.20 ***	0.12 *	0.40 ***	0.03 ***	0.04	0.14 **	0.04	0.27 ***	-

Note. * *p* < 0.05; ** *p* < 0.01; *** *p* < 0.001

## Data Availability

The raw data supporting the conclusions of this article will be made available by the authors on request.

## Supplementary Materials

The following supporting information can be downloaded at: https://www.mdpi.com/article/10.3390/ejihpe16020019/s1, Supplementary File S1: Table S1. Edge weight matrix from the EBICglasso network; Table S2. Centrality measures per variable using the EBICglasso estimator; Supplementary File S2: Table S1. Gender Edge weight matrix from the EBICglasso network; Table S2. Gender Centrality measures per variable using the EBICglasso estimator; Table S3. Edge weight matrix from the partial correlation network; Table S4. Centrality measures per variable based on the partial correlation estimator.
