# Supplementary material for: A Network Analysis of Inner Strength Among University Students with Borderline Personality Disorder Symptoms
Source: Eur J Investig Health Psychol Educ. 2026 Jan 31;16(2):19. doi: 10.3390/ejihpe16020019 (PMC12939644; doi:10.3390/ejihpe16020019)
Supplement: Supplementary file 1 [file ejihpe-16-00019-s001.zip › Supplementary File S1, jan 7.pdf]

Table S1. Edge weight matrix from the EBICglasso network

	Truthfulness	Perseverance	Wisdom	Generosity	Five-Precepts	Meditation	Tolerance	Equanimity	Determination	Loving-kindness
Truthfulness	-									
Perseverance	0.187	-								
Wisdom	0.068	0.318	-							
Generosity	0.185	0.192	0.265	-						
Five-Precepts	0.094	0.217	0.233	0.209	-					
Meditation	0.082	0.221	0.145	0.073	0.245	-				
Tolerance	0.182	0.178	0.24	0.282	0.103	0.057	-			
Equanimity	-0.069	-0.021	0.146	0.139	0.196	0.004	0.223	-		
Determination	0.088	0.23	0.295	0.207	0.238	0.251	0.167	0.182	-	
Loving-kindness	0.18	0.197	0.123	0.399	0.251	0.039	0.143	0.04	0.265	-

Table S2. Centrality measures per variable using the EBICglasso estimator

Variables	Betweenness	Closeness	Strength	Expected Influence
Truthfulness	-1.594	0.000	0.656	0.346
Perseverance	-0.266	0.011	0.931	0.715
Wisdom	0.399	0.012	0.958	0.746
Generosity	-0.266	0.013	0.897	0.897
Five-Precepts	0.399	0.012	0.904	0.822
Meditation	-0.93	0.010	0.723	0.407
Tolerance	-0.93	0.011	0.799	0.709
Equanimity	1.727	0.012	0.974	0.279
Determination	1.063	0.012	0.882	0.882
Loving-kindness	0.399	0.013	1.069	0.635
