# Supplementary material for: A Network Analysis of Inner Strength Among University Students with Borderline Personality Disorder Symptoms
Source: Eur J Investig Health Psychol Educ. 2026 Jan 31;16(2):19. doi: 10.3390/ejihpe16020019 (PMC12939644; doi:10.3390/ejihpe16020019)
Supplement: Supplementary file 1 [file ejihpe-16-00019-s001.zip › Supplementary File S2, jan 7.pdf]

Table S1. Edge weight matrix from the EBICglasso network

	Female										Male									
**Variable**	**SBI1**	**SBI2**	**SBI3**	**SBI4**	**SBI5**	**SBI6**	**SBI7**	**SBI8**	**SBI9**	**SBI10**	**SBI1**	**SBI2**	**SBI3**	**SBI4**	**SBI5**	**SBI6**	**SBI7**	**SBI8**	**SBI9**	**SBI10**
SBI1																				
SBI2	0.056										0.000									
SBI3	0.000	0.245									0.000	0.000								
SBI4	0.131	0.058	0.010								0.000	0.000	0.000							
SBI5	0.000	0.101	0.046	0.000							0.000	0.000	0.000	0.000						
SBI6	0.000	0.242	0.000	0.000	0.224						0.000	0.000	0.000	0.000	0.000					
SBI7	0.175	0.095	0.052	0.120	0.028	0.000					0.000	0.000	0.000	0.000	0.000	0.000				
SBI8	0.000	0.000	0.000	0.000	0.153	0.000	0.143				0.000	0.000	0.000	0.000	0.000	0.000	0.000			
SBI9	0.000	0.040	0.130	0.015	0.019	0.249	0.026	0.094			0.000	0.000	0.000	0.000	0.000	0.000	0.000	0.000		
SBI10	0.000	0.061	0.000	0.327	0.063	0.000	0.000	0.000	0.144		0.000	0.000	0.000	0.000	0.000	0.000	0.000	0.000	0.000	

Note. All indices were standardized (z scores) within each network. Networks were estimated using the EBICglasso procedure. In the male subsample, all centrality indices were estimated as zero due to the absence of non-zero edges after regularization.

Table S2. Centrality measures per variable using the EBICglasso estimator

	Female				Male			
**Variable**	**Betweenness**	**Closeness**	**Strength**	**Expected influence**	**Betweenness**	**Closeness**	**Strength**	**Expected influence**
SBI1	-1.346	-1.692	-1.529	-1.529	0.000	0.000	0.000	0.000
SBI10	0.256	0.037	-0.093	-0.093	0.000	0.000	0.000	0.000
SBI2	0.577	0.637	1.785	1.785	0.000	0.000	0.000	0.000
SBI3	-1.346	-0.933	-0.781	-0.781	0.000	0.000	0.000	0.000
SBI4	-0.385	-0.444	0.323	0.323	0.000	0.000	0.000	0.000
SBI5	-0.705	-0.105	0.150	0.150	0.000	0.000	0.000	0.000
SBI6	1.218	1.400	0.653	0.653	0.000	0.000	0.000	0.000
SBI7	0.577	0.237	0.182	0.182	0.000	0.000	0.000	0.000
SBI8	-0.385	-0.632	-1.353	-1.353	0.000	0.000	0.000	0.000
SBI9	1.539	1.495	0.664	0.664	0.000	0.000	0.000	0.000

Table S3. Edge weight matrix from the partial correlation network

Female	Male

	I1	I2	I3	I4	I5	I6	I7	I8	I9	I10	I1	I2	I3	I4	I5	I6	I7	I8	I9	I10
I1																				
I2	0.074										0.329									
I3	-0.006	0.295									-0.101	0.208								
I4	0.158	0.056	0.07								-0.027	0.007	0.426							
I5	-0.035	0.12	0.085	-0.008							0.115	0.016	0.084	0.143						
I6	0.016	0.28	-0.059	-0.02	0.272						0.168	-0.06	0.27	-0.193	0.316					
I7	0.24	0.124	0.065	0.141	0.037	0.008					0.004	0.073	0.205	0.236	-0.281	0.084				
I8	-0.109	-0.117	0.009	0.039	0.228	-0.056	0.226				-0.082	-0.133	0.123	0.155	0.153	-0.115	0.194			
I9	0.009	0.028	0.186	0.012	-0.015	0.307	0.026	0.162			0.101	0.111	-0.078	0.177	0.213	0.117	0.12	0.119		
I10	0.034	0.118	-0.114	0.375	0.11	-0.051	-0.045	0.013	0.198		0.054	0.141	-0.131	0.129	0.293	-0.103	0.128	-0.086	0.226	

Note. I1 = Truthfulness; I2 = Perseverance; I3 = Wisdom; I4 = Generosity; I5 = Five-Precepts; I6 = Meditation; I7 = Tolerance; I8 = Equanimity; I9 = Determination; I10 = Loving-Kindness.

Table S4. Centrality measures per variable based on the partial correlation estimator

	Female				Male			
**Variable**	**Betweenness**	**Closeness**	**Strength**	**Expected influence**	**Betweenness**	**Closeness**	**Strength**	**Expected influence**
SBI1	-1.399	-2.114	-1.920	-1.524	-0.752	-1.701	-1.580	-0.763
SBI10	1.144	0.492	0.760	-0.286	-0.334	-0.235	-0.159	-0.438
SBI2	1.144	0.888	1.853	1.351	0.919	-0.630	-1.141	-0.283
SBI3	-1.399	-0.972	-0.432	-0.801	0.919	1.193	1.389	0.866
SBI4	-0.127	-0.506	-0.512	0.606	-0.334	0.897	0.765	1.040
SBI5	-0.975	0.073	-0.292	0.465	2.172	1.154	1.331	1.038
SBI6	0.297	0.501	0.829	0.002	-0.334	0.835	0.466	-1.043
SBI7	1.144	0.027	-0.271	0.602	-0.752	0.062	-0.005	-0.026
SBI8	-0.127	0.197	0.044	-1.452	-0.752	-1.065	-0.766	-1.622
SBI9	0.297	1.415	-0.059	1.036	-0.752	-0.509	-0.298	1.231
